# Broad Coverage Precoding for 3D Massive MIMO with Huge Uniform Planar Arrays

**DOI:** 10.3390/e23070887

**Published:** 2021-07-13

**Authors:** An-An Lu, Yan Chen, Xiqi Gao

**Affiliations:** 1 National Mobile Communications Research Laboratory (NCRL), Southeast University, Nanjing 210096, China; aalu@seu.edu.cn (A.-A.L.); 213160372@seu.edu.cn (Y.C.); 2 Purple Mountain Laboratories, Nanjing 211111, China

**Keywords:** massive multi-input multi-output (MIMO), broad coverage precoder design, uniform planar array (UPA), high dimension

## Abstract

In this paper, we propose a novel broad coverage precoder design for three-dimensional (3D) massive multi-input multi-output (MIMO) equipped with huge uniform planar arrays (UPAs). The desired two-dimensional (2D) angle power spectrum is assumed to be separable. We use the per-antenna constant power constraint and the semi-unitary constraint which are widely used in the literature. For normal broad coverage precoder design, the dimension of the optimization space is the product of the number of antennas at the base station (BS) and the number of transmit streams. With the proposed method, the design of the high-dimensional precoding matrices is reduced to that of a set of low-dimensional orthonormal vectors, and of a pair of low-dimensional vectors. The dimensions of the vectors in the set and the pair are the number of antennas per column and per row of the UPA, respectively. We then use optimization methods to generate the set of orthonormal vectors and the pair of vectors, respectively. Finally, simulation results show that the proposed broad coverage precoding matrices achieve nearly the same performance as the normal broad coverage precoder with much lower computational complexity.

## 1. Introduction

Massive multiple-input multiple-output (MIMO) [1,2] is one of the key enabling technologies of fifth generation (5G) wireless communications systems. It provides huge spatial multiplexing gains and high energy efficiency by employing a large number of antennas at a base station (BS). In the process of establishing links between the BS and the users, the broadcasting and control information plays an important role. In practical systems, uniform planar array (UPA) antennas are preferred to uniform linear array (ULA) antennas due to their compact size. In this paper, we focus on the broad coverage precoder design for synchronization and control information transmission in a massive MIMO equipped with UPAs.

### 1.1. Existing Works

For massive MIMO systems equipped with ULAs, omnidirectional precoding has been proposed in the literature [3,4,5] recently. It is designed by assuming the channel state information (CSI) is unavailable. In [3], the received mean signal power needed to be constant at finite discrete angles under the per-antenna power constraint and the semi-unitary constraint. In [4,5], the space-time codes are used to design a precoder for omnidirectional transmission at any time. The received signal power is designed to be constant at finite discrete angles in [4] and any angle in [5].

In omnidirectional precoder design for massive MIMO systems with ULAs, Golay complementary sequences play a key role. These sequences was introduced by Golay [6] in 1951. The pair of complementary sequences was then generalized to a set of complementary sequences [7], which are also called Welti codes [8]. Furthermore, Golay complementary sequences are generalized to Golay complementary arrays [9,10] or high-dimensional Welti codes, which have been investigated in [11,12]. Inspired by the Golay complementary arrays and the high-dimensional Welti codes, a novel and low-complexity array design is proposed in [13] to construct the omnidirectional precoding matrices for massive MIMO with UPAs. Omnidirectional precoder design for massive MIMO with UPAs has also been investigated in [14,15,16] based on complementary codes, numerical optimization methods and Golay complementary matrices.

In practical systems, omnidirectional precoding might cause interference with other cells or sectors. To solve this problem, broad coverage precoding is proposed in [17]. It is designed for massive MIMO with ULAs based on manifold optimization under the per-antenna constant power and semi-unitary constraints. By considering the synchronization performance, the work in [17] is then extended to [18,19] for broad coverage precoder design for three-dimensional (3D) massive MIMO in terrestrial and satellite communication systems, respectively. However, as the size of antennas is still increasing, the complexity of the broad coverage precoder design for massive MIMO with a large number of antennas will be a problem. For example, the optimization in [18] is performed over the space of multiple matrices and thus will be too complicated for huge antenna arrays.

### 1.2. Main Contribution

In this paper, we propose a broad coverage precoder design for 3D massive MIMO with huge UPAs. The two-dimensional (2D) angle power spectrum for the broad coverage precoding is assumed to be separable. We also use the per-antenna power constraint and the semi-unitary constraint. The number of antennas equipped at the BS is $M_{t}=M_{z}M_{x}$. The number of transmit streams is *r*. With the proposed design method, the design of the high-dimensional $M_{t}\times r$ precoding matrices is reduced to that of a pair of low-dimensional $M_{x}\times 1$ vectors, and of a set with length *r* of low-dimensional $M_{z}\times 1$ orthonormal vectors.

The main contributions of the paper are summarized as follows: Theorem 1 provides a method to generate high-dimensional precoding matrices with a separate 2D angle power spectrum from low-dimensional vectors. Theorem 2 proves the conditions for low-dimensional vectors to generate high-dimensional precoding matrices satisfying the per-antenna power constraint. Based on Theorems 1 and 2, a low-complexity broad coverage precoder design is proposed. Furthermore, manifold optimization is used to generate a set of orthonormal vectors with the required angle power spectrum. The design of two vectors with nonzero elements with a constant envelope and required angle power spectrum is also provided.

The rest of the paper is organized as follows. The system model and problem formulation are provided in Section 1. The proposed method for designing 2D broad coverage precoding matrices is given in Section 3. Simulation results are presented in Section 4. The conclusion is drawn in Section 5. A lemma and proofs of theorems are in the Appendix.

*Notations*: Throughout this paper, lowercase and uppercase boldface letters are used to denote column vectors and matrices, respectively. The superscripts ${\left(\cdot \right)}^{*}$, ${\left(\cdot \right)}^{T}$ and ${\left(\cdot \right)}^{H}$ indicate the conjugate, transpose and conjugate transpose operation, respectively. The operator ⊗ is used to denote the Kronecker product. The notation tr(·) represents the trace function of a matrix. The operator diag(A) represents the diagonal matrix obtained by using the diagonal elements of A. The identity matrix with dimension N×N is denoted by $I_{N}$.

## 2. System Model and Problem Formulation

### 2.1. System Model

We consider a 3D massive MIMO system where a UPA is equipped at a BS. The number of antennas in the antenna array is $M_{t}=M_{z}M_{x}$, where $M_{z}$ and $M_{x}$ denote the numbers of antennas in each row and column, respectively. In this paper, we focus on the problem of broad coverage precoder design for 3D massive MIMO systems. We define h as the $M_{t}\times 1$ channel vector from the BS to a point P=(ρ,θ,ϕ) at the free space, where ρ denotes the distance between the locations of the BS and the point *P*, and θ and ϕ represent the polar and azimuthal angles, respectively. We assume *r* transmitted data streams are used in precoding. At the point *P*, the received signal can be written as

(1) y=hPx+z,

where the symbol P denotes the $M_{t}\times r$ precoding matrix, x is the r×1 transmitted vector and *z* is a complex Gaussian noise variable. The mean and variance of *z* is 0 and $\sigma_{z}^{2}$. Let $p_{i}$ denote the vector by extracting the *i*-th column of P. We reshape the vector $p_{i}$ into a matrix of $C^{M_{z}\times M_{x}}$ and use $P_{i}$ to denote it. Then, the matrix P can be re-expressed as

(2) $$P=\left[p_{1}\;p_{2}\;\cdots \;p_{r}\right]=\left[\mathrm{vec}\left(P_{1}\right)\;\mathrm{vec}\left(P_{2}\right)\;\cdots \;\mathrm{vec}\left(P_{r}\right)\right].$$

### 2.2. Problem Formulation

In practical massive MIMO systems, the precoder for public information transmission should be specifically designed to provide realistic broad coverage. The required 2D angle power spectrum in the free space is usually given according to the system parameters.

Let U(θ,ϕ) be the steering matrix in the direction (θ,ϕ) with the elements being defined as

(3) $${\left[U\left(\theta ,\phi \right)\right]}_{kl}=e^{-\left(k-1\right)\frac{2j\pi d_{z}\cos\theta }{\lambda }}e^{-\left(l-1\right)\frac{2j\pi d_{x}\sin\theta \cos\phi }{\lambda }},$$

where $k=1,2,\cdots ,M_{z}$ and $l=1,2,\cdots ,M_{x}$. The spacings $d_{z}$ and $d_{x}$ are assumed to be equal to 0.5λ. For convenience, we set $u=\frac{1}{2}\cos\theta$ and $v=\frac{1}{2}\sin\theta \cos\phi$ as the scaled directional cosines with respect to the *z* and *x* axes, respectively. Then, the steering matrix U(θ,ϕ) in Equation (3) can be rewritten as

(4) $$U\left(u,v\right)=v_{z}\left(u\right)⊗v_{x}{\left(v\right)}^{T},$$

where $v_{z}\left(u\right)$ and $v_{x}\left(v\right)$ are the steering vectors in vertical and horizontal directions, defined by

(5) $$v_{z}\left(u\right)=\frac{1}{\sqrt{M_{z}}}{\left[1\;\;e^{-j2\pi u}\;\;\;\;\cdots \;\;e^{-(M_{z}-1)j2\pi u}\right]}^{T}$$

(6) $$v_{x}\left(v\right)=\frac{1}{\sqrt{M_{x}}}{\left[1\;\;e^{-j2\pi v}\;\;\;\;\cdots \;\;e^{-(M_{x}-1)j2\pi v}\right]}^{T}.$$

Let a(u,v) be the steering vector obtained by vectorizing the steering matrix U(u,v) as

(7) u(u,v)=vec(U(u,v)).

Then, we have

(8) $$u\left(u,v\right)=v_{x}\left(v\right)⊗v_{z}\left(u\right).$$

We assume the desired 2D angle power spectrum for broad coverage is separable in u,v, i.e., the 2D angle power spectrum to the angle (u,v) is given by

(9) $$\sum_{i=1}^{r}{\left|u{\left(u,v\right)}^{T}\mathrm{vec}\left(P_{i}\right)\right|}^{2}=a_{z}\left(u\right)a_{x}\left(v\right),$$

where $a_{z}\left(u\right)$ and $a_{x}\left(v\right)$ denote the functions of the scaled directional cosines *u* and *v*. Substituting Equation (8) into Equation (9), we then obtain

(10) $$\sum_{i=1}^{r}{\left|v_{z}{\left(u\right)}^{T}P_{i}v_{x}\left(v\right)\right|}^{2}=a_{z}\left(u\right)a_{x}\left(v\right).$$

For brevity, we define

(11) $$s_{P_{i}}\left(u,v\right)={\left|v_{z}{\left(u\right)}^{T}P_{i}v_{x}\left(v\right)\right|}^{2}$$

then we have

(12) $$\sum_{i=1}^{r}s_{P_{i}}\left(u,v\right)=a_{z}\left(u\right)a_{x}\left(v\right).$$

After discussing the desired 2D angle power spectrum, we need to consider the constraints that the precoding matrices need to satisfy. To maximize the power efficiency of all the antennas, a commonly used constraint in the literature is the per-antenna constant power constraint. Let $p_{i,kl}$ denote the k,l-th element in the matrix $P_{i}$. We formulate this constraint as

(13) $$\sum_{i=1}^{r}{\left|p_{i,kl}\right|}^{2}=1,\;\forall k,l.$$

For a public channel, the CSI is usually not known. To maximize the mutual information when the channel vector h has independent and identically distributed (i.i.d.) entries with zero mean and unit variance [20], we then consider the semi-unitary constraint

(14) $$P^{H}P=\frac{M_{t}}{r}I_{r}.$$

In conclusion, the broad coverage precoding matrices need to be designed by using the following three conditions:

(15) $$\sum_{i=1}^{r}s_{P_{i}}\left(u,v\right)=a_{z}\left(u\right)a_{x}\left(v\right)$$

(16) $$\sum_{i=1}^{r}{\left|p_{i,kl}\right|}^{2}=1,\;\forall k,l$$

(17) $$P^{H}P=\frac{M_{t}}{r}I_{r}.$$

When $a_{z}\left(u\right)=1$ and $a_{x}\left(v\right)=1$, the problem is reduced to that for omnidirectional precoder design in [13]. In this paper, we aim to solve the problem for general $a_{z}\left(u\right)$ and $a_{x}\left(v\right)$.

## 3. Broad Coverage Precoding Matrix Design for UPAs

In this section, we propose the design of the broad coverage precoder for 3D massive MIMO with UPAs.

### 3.1. Preliminary Background

Before presenting the broad coverage precoder design, we give some definitions which will be used in the paper. Let $D=\{d_{1},d_{2},\cdots ,d_{r}\}$ and $F=\{f_{1},f_{2},\cdots ,f_{r}\}$ be two sets of $M_{z}\times 1$ vectors, respectively. Their aperiodic cross-correlation is defined as

(18) $$c_{D,F}\left(t\right)=\sum_{i=1}^{r}\sum_{j=1}^{M_{z}-t}{\left[d_{i}\right]}_{j}{\left[f_{i}\right]}_{j+t}^{*}=c_{F,D}{\left(-t\right)}^{*},$$

where $t=0,1,\cdots ,M_{z}-1$. For convenience, we define the angle power spectrum of column vectors and row vectors in a different way. The angle power spectrum of a column vector $d_{i}$ is defined as

(19) $$s_{d_{i}}\left(u\right)={\left|v_{z}{\left(u\right)}^{T}d_{i}\right|}^{2}.$$

The angle power spectrum of a row vector ϕ is defined as

(20) $$s_{\phi }\left(v\right)={\left|\phi^{T}v_{x}\left(v\right)\right|}^{2}.$$

Let a denote a vector ${\left[a_{1},a_{2},\cdots ,a_{n}\right]}^{T}$ and $a^{†}$ denote its complex conjugate of the reversal, i.e., $a^{†}={\left[a_{n}^{*}\;a_{n-1}^{*}\;\cdots \;a_{1}^{*}\right]}^{T}$.

### 3.2. Broad Coverage Precoder Design

If we optimize P directly, we need to search over an $rM_{z}M_{x}$ dimensional space. For massive MIMO, $rM_{z}M_{x}$ is usually very large and is still increasing as wireless communications systems evolve, thus the complexity of the optimization will be very high. To reduce the computational complexity, we need to find certain special structures that can be employed to transform the optimization problem with high dimensions into one with low dimensions. In [13], an omnidirectional precoder design for 3D massive MIMO with UPAs is proposed. Among the many theorems, Theorem 1 in [13] is an easy, but important, one. It will also play an important role in helping us to transform the optimization problem with high dimensions into one with low dimensions. Thus, we present it in Appendix A as Lemma A1.

With Lemma A1, we can prove the following theorem, which can be used to generate the precoding matrices having the first property in Equation (15) required by broad coverage precoding.

**Theorem** **1.** *Let the two sets D and F of $M_{z}\times 1$ vectors be defined as $D=\{d_{1},d_{2},\cdots ,d_{r}\}$ and $F=\{f_{1},f_{2},\cdots ,f_{r}\}$ with*

(21) $$\begin{array}{c} c_{D,F}\left(t\right)=0,t=-M_{z}+1,\cdots ,M_{z}-1. \end{array}$$

*Furthermore, the sums of the angle power spectrum of the vectors in these two sets are equal, and are given as*

(22) $$\begin{array}{c} \sum_{i=1}^{r}s_{d_{i}}\left(u\right)=\sum_{i=1}^{r}s_{f_{i}}\left(u\right)=a_{z}\left(u\right). \end{array}$$

*Let **ϕ** and **φ** denote two $M_{x}\times 1$ vectors with the sum of their angle power spectrum being given by*

(23) $$s_{\phi }\left(v\right)+s_{\varphi }\left(v\right)=a_{x}\left(v\right).$$

*The precoding matrices $P_{i}$ are defined as*

(24) $$\begin{array}{c} P_{i}=d_{i}⊗\phi^{T}+f_{i}⊗\varphi^{T},\;i=1,2,\cdots ,r. \end{array}$$

*Then, the sum of the 2D angle power spectrum of these precoding matrices is*

(25) $$\begin{array}{cc} & \sum_{i=1}^{r}s_{P_{i}}\left(u,v\right)=a_{z}\left(u\right)a_{x}\left(v\right). \end{array}$$

**Proof.**  The proof is provided in Appendix B. □

Theorem 1 provides a method to generate high-dimensional precoding matrices having the first condition required by broad coverage precoding from low-dimensional vectors. When D and F are both sets of orthonormal vectors, it can be easily proved that the third condition required by broad coverage precoding is also satisfied. Thus, we only need to let the precoding matrices satisfy the second condition. We then obtain a sufficient condition for the precoding matrices to satisfy the second condition in the following theorem.

**Theorem** **2.** *Let D and F be two sets of $M_{z}\times 1$ vectors defined as $D=\{d_{1},d_{2},\cdots ,d_{r}\}$ and $F=\{f_{1},f_{2},\cdots ,f_{r}\}$ with*

(26) $$\begin{array}{c} \sum_{i=1}^{r}{\left|d_{ik}\right|}^{2}=1,\;k=1,2,\cdots ,M_{z} \end{array}$$

(27) $$\begin{array}{c} \sum_{i=1}^{r}{\left|f_{ik}\right|}^{2}=1,\;k=1,2,\cdots ,M_{z}, \end{array}$$

*where $d_{ik}$ and $f_{ik}$ denote the k-th elements of $d_{i}$ and $f_{i}$, respectively. Let **α** and **β** be two vectors with binary entries and $\alpha_{j}+\beta_{j}=1$, $j=1,2,\cdots ,M_{x}$. Let **ϕ** and **φ** be two $M_{x}\times 1$ vectors defined by ${\left[\phi \right]}_{j}=e^{-j2\pi w_{j}}\alpha_{j}$ and ${\left[\varphi \right]}_{j}=e^{-j2\pi w_{j}}\beta_{j}$. The precoding matrices $P_{i}$ are defined as*

(28) $$\begin{array}{c} P_{i}=d_{i}⊗\phi^{T}+f_{i}⊗\varphi^{T},\;i=1,2,\cdots ,r. \end{array}$$

*Then, we have*

(29) $$\sum_{i=1}^{r}{\left|p_{i,kl}\right|}^{2}=1,\;\forall k,l.$$

**Proof.**  The proof is provided in Appendix C. □

Theorem 2 has been presented as part of Theorem 3 in [13]. However, putting it in an independent theorem makes it easier to understand what is the condition for the per-antenna constant power constraint. We define $D=[d_{1}\;d_{2}\;\cdots \;d_{r}]$ and $F=[f_{1}\;f_{2}\;\cdots \;f_{r}]$. The conditions in Equations (26) and (27) are equivalent to that the matrices D and F should satisfy the constraints

(30) $$\mathrm{diag}\left(DD^{H}\right)=I_{M_{z}}$$

and

(31) $$\mathrm{diag}\left(FF^{H}\right)=I_{M_{z}}.$$

The conditions for ϕ and φ in Theorem 2 can be rephrased as follows. The nonzero elements in ϕ and φ have a constant envelope, and their locations in ϕ and φ are complementary.

Combining Theorems 1 and 2, we can obtain a broad coverage precoder design, which is a generalized version of Theorem 3 in [13] for omnidirectional precoder design. When ϕ and φ are a pair of complementary sequences, and D and F are two sets of orthonormal complementary vectors, the combination of Theorems 1 and 2 reduces to Theorem 3 in [13]. From Theorems 1 and 2, we see that the broad coverage precoding matrices can be obtained from the pair ϕ and φ and the two sets D and F. Thus, instead of optimizing the high-dimensional precoding matrices directly, we can optimize the low-dimensional ϕ and φ, and the low-dimensional $d_{1},d_{2},\cdots ,d_{r}$ and $f_{1},f_{2},\cdots ,f_{r}$, respectively.

In the following, we first investigate the design of D and F having zero aperiodic cross-correlation. Theorem 11 in [7] presents a method to construct two sets having zero cross-correlation when *r* is even. The method is used to generate sets of complementary vectors with bipolar elements, but can be extended to design sets of vectors with arbitrary elements easily, as shown in the following theorem.

**Theorem** **3.** *Let $D=\{d_{1},d_{2},\cdots ,d_{r}\}$ be a set of vectors, where r is even. Let $F=\{f_{1},f_{2},\cdots ,f_{r}\}$ be a set of vectors defined by $f_{1}=d_{2}^{†}$, $f_{2}=-d_{1}^{†}$, $f_{3}=d_{4}^{†}$, $f_{4}=-d_{3}^{†}$, $\cdots ,f_{r}=-d_{r-1}^{†}$. Then, we have*

(32) $$\begin{array}{c} c_{D,F}\left(t\right)=0,t=-M_{z}+1,\cdots ,M_{z}-1. \end{array}$$

**Proof.**  The proof is similar to that in [7] and is omitted here for brevity. □

Theorem 3 presents a method to obtain the set F whose aperiodic cross-correlation with the set D is zero when the latter is already given. We can also observe that the sums of the angle power spectrum of these two sets are the same, and that the vectors in F are also orthonormal vectors if the vectors in D are orthonormal vectors. Thus, instead of designing two sets, we only need to design one set of orthonormal vectors with the required angle power spectrum.

### 3.3. Design of Set D

In this subsection, we introduce the design of the set D for broad coverage precoder design. Since $D=\{d_{1},d_{2},\cdots ,d_{r}\}$ is a set of orthonormal column vectors, we have

(33) $$D^{H}D=\frac{M_{z}}{r}I_{r}.$$

From Equation (22), we then see that D should also satisfy

(34) $$v_{z}{\left(u\right)}^{T}DD^{H}v_{z}{\left(u\right)}^{*}=a_{z}\left(u\right).$$

To find the matrix D that satisfies the above equation, we formulate the problem as

(35) $$\begin{array}{cc} \mathrm{find}\;\; & D\\ \;\;s.t.\; & v_{z}{\left(u\right)}^{T}DD^{H}v_{z}{\left(u\right)}^{*}=a_{z}\left(u\right).\\ \; & \mathrm{diag}\left(DD^{H}\right)=I_{M_{z}}\\ \; & D^{H}D=\frac{M_{z}}{r}I_{r}. \end{array}$$

It is very hard to find D that satisfies Equation (35) directly because

$$v_{z}{\left(u\right)}^{T}DD^{H}v_{z}{\left(u\right)}^{*}=a_{z}\left(u\right)$$

might have no solution. To make the optimization problem valid, we sample the function $a_{z}\left(u\right)$ at the points

$$u_{k}=\frac{k}{N_{z}M_{z}},k=1,2,\cdots ,N_{z}M_{z},$$

where $N_{z}$ is the sampling ratio. Let $a_{z,k}=a_{z}\left(u_{k}\right)$. Let $V_{z}^{F}$ be an $N_{z}M_{z}\times N_{z}M_{z}$ DFT matrix, and $V_{z}$ be an $N_{z}M_{z}\times M_{z}$ matrix defined by

(36) $$V_{z}=V_{z}^{F}\left(\begin{array}{c} I_{M_{z}}\\ 0_{\left(N_{z}-1\right)M_{z}} \end{array}\right).$$

We then try to find an approximate solution of

$$e_{k}^{T}V_{z}DD^{H}V_{z}^{H}e_{k}=a_{z,k},k=1,2,\cdots ,N_{z}M_{z}$$

under the other constraints. To achieve this, we need to define a distance or divergence between the sequences $e_{k}^{T}V_{z}DD^{H}V_{z}^{H}e_{k}$ and $a_{z,k}$. Let ${\hat{a}}_{z,k}$ be defined as

(37) $${\hat{a}}_{z,k}=e_{k}^{T}V_{z}DD^{H}V_{z}^{H}e_{k},k=1,2,\cdots ,N_{z}M_{z}.$$

Let $a_{z}$ and ${\hat{a}}_{z}$ denote the sequences $\left\{a_{z,k}\right\}$ and $\left\{{\hat{a}}_{z,k}\right\}$, respectively. The KL divergence between the sequences $a_{z}$ and ${\hat{a}}_{z}$ is defined as [21]

(38) $$\begin{array}{cc} \; & D_{KL}\left(a_{z}:{\hat{a}}_{z}\right)\\ \; & =\sum_{k=1}^{N_{z}M_{z}}a_{z,k}\log\frac{a_{z,k}}{{\hat{a}}_{z,k}}-\sum_{k=1}^{N_{z}M_{z}}a_{z,k}+\sum_{k=1}^{N_{z}M_{z}}{\hat{a}}_{z,k}. \end{array}$$

From the definition of ${\hat{a}}_{z,k}$ and $V_{z}^{H}V_{z}=I_{M_{z}}$, we have

(39) $$\begin{array}{cc} \sum_{k=1}^{N_{z}M_{z}}{\hat{a}}_{z,k} & =\mathrm{tr}(V_{z}DD^{H}V_{z}^{H})\\ \; & =\mathrm{tr}(DD^{H}V_{z}^{H}V_{z})\\ \; & =\mathrm{tr}(DD^{H})\\ \; & =M_{z}. \end{array}$$

Then, the KL divergence can be rewritten as

(40) $$\begin{array}{cc} D_{KL}\left(a_{z}:{\hat{a}}_{z}\right) & =\sum_{k=1}^{N_{z}M_{z}}-a_{z,k}\log{\hat{a}}_{z,k}+c_{k}, \end{array}$$

where

(41) $$c_{k}=\sum_{k=1}^{N_{z}M_{z}}a_{z,k}\log a_{z,k}-\sum_{k=1}^{N_{z}M_{z}}a_{z,k}+M_{z}$$

is a constant not related to D.

By using the KL divergence, we define $f\left(D\right)=D_{KL}\left(a_{z}:{\hat{a}}_{z}\right)$. We then formulate an optimization problem approximate to that in Equation (35) as

(42) $$\begin{array}{cc} \min_{D} & f(D)\\ \;\;s.t. & \mathrm{diag}\left(DD^{H}\right)=I_{M_{z}}\\ & D^{H}D=\frac{M_{z}}{r}I_{r}. \end{array}$$

The constrained optimization problem (42) is very hard to handle since its constraints are not as simple as linear or quadratic constraints. For such problems, manifold optimization is a powerful alternative [22]. It has already been widely used in MIMO systems to handle various complicated constraints. The most commonly addressed manifolds in the MIMO literature are Grassmann and Stiefel manifolds [23,24,25,26]. For the optimization problem (42), the two constraints are the Stiefel manifold

(43) $$U=\{D\in C^{M_{z}\times r}|D^{H}D=\frac{M_{z}}{r}I_{r}\}$$

and the oblique manifold [27]

(44) $$O=\{D\in C^{M_{z}\times r}|\mathrm{diag}\left(DD^{H}\right)=I_{M_{z}}\}.$$

The optimization problem is then reformulated as

(45) $$\begin{array}{cc} \arg\min_{D} & f(D)\\ s.t. & D\in O\cap U. \end{array}$$

The obtained optimization problem is over the intersection of two manifolds. The problem (45) can also be used to design the broad coverage precoder for ULAs, which has been investigated in [17] with a different objective function based on manifold optimization. It has also been investigated in [13] with f(D) being changed to the objective function for omnidirectional precoding. Thus, it can also be solved by using the proposed methods in [13,17]. Since the projected gradient method on the intersection of two manifolds provided in [13] is simpler, we also use it in this work.

In the following, we briefly introduce the projected gradient method. The gradient of the objective function *f* in the Euclidean space is obtained as

(46) $$\nabla f\left(D\right)=\frac{\partial f(D)}{\partial D^{*}}=\sum_{k=1}^{N_{z}M_{z}}-a_{z,k}{\hat{a}}_{z,k}^{-1}V^{H}e_{k}e_{k}^{T}VD.$$

With the obtained Euclidean gradient, we can search along the gradient direction ∇f(D) to find the optimal D. Let μ be the step size. After moving a step ahead, the obtained point D−μ∇f(D) is out of O∩U. We need to project it back to O∩U. To achieve this, we first define the projections onto the manifolds O and U as

(47) $$P_{O}\left(D\right)={\left(\mathrm{diag}\left(DD^{H}\right)\right)}^{-1/2}D$$

and

(48) $$P_{U}\left(D\right)=\sqrt{\frac{M_{z}}{r}}D{\left(D^{H}D\right)}^{-1/2}.$$

From [28], the projection onto O∩U is obtained as

(49) $$P_{O\cap U}\left(D\right)=P_{O}\left(P_{U}\left(\cdots P_{O}\left(P_{U}\left(D\right)\right)\right)\right),$$

where ⋯ indicates that an infinite number of projections are needed theoretically. In practical use, the alternative projections proceed until reaching a pre-set target. By using the projection, the point D−μ∇f(D) can be projected back to the intersection of two manifolds O∩U by $P_{O\cap U}\left(D-\mu \nabla f\left(D\right)\right)$.

The projected gradient method is summarized as

(50) $$D^{d+1}=P_{O\cap U}\left(D^{d}-\mu \nabla f\left(D^{d}\right)\right),$$

where $D^{d}$ is the point obtained after d−1 iterations. To guarantee the convergence, μ should have a very small value or needs to be determined by the line search method [22]. By using the projected gradient method, an optimal point of the problem (45) is obtained.

### 3.4. Design of the Pair

In this subsection, we investigate the design of ϕ and φ. To meet the condition provided in Theorem 2, the pair ϕ and φ should satisfy

(51) $$s_{\phi }\left(v\right)+s_{\varphi }\left(v\right)=a_{x}\left(v\right)$$

and their elements should satisfy $\phi_{j}=e^{-j2\pi w_{j}}\alpha_{j}$ and $\varphi_{j}=e^{-j2\pi w_{j}}\beta_{j}$, where $\alpha_{j}$ and $\beta_{j}$ are binary and $\alpha_{j}+\beta_{j}=1$. For simplicity, we consider the case when $M_{x}$ is even and fix the structures of ϕ and φ in this paper. We redefine ϕ and φ as

(52) $$\phi ={\left[e^{-j2\pi \rho_{1}}\;e^{-j2\pi \rho_{2}}\;\cdots \;e^{-j2\pi \rho_{M_{x}/2}}\;0\;\cdots \;0\right]}^{T}$$

and

(53) $$\varphi ={\left[0\;\cdots \;0\;e^{-j2\pi \lambda_{1}}\;e^{-j2\pi \lambda_{2}}\;\cdots \;e^{-j2\pi \lambda_{M_{x}/2}}\right]}^{T}.$$

It can be observed that the condition needed for the vectors ϕ and φ to ensure the per-antenna constant power constraint is still satisfied. Instead of optimizing ϕ and φ directly, we will optimize $\rho_{i}$ and $\lambda_{i}$, $i=1,2,\cdots ,M_{x}/2$ since they have no constraint.

It is also very hard to obtain $\rho_{i}$ and $\lambda_{i}$ that satisfy Equation (51) because it might have no solution. To obtain a target than can make the optimization easier, we sample the $a_{x}\left(v\right)$ in the points

$$v_{k}=\frac{k}{N_{x}M_{x}},k=1,2,\cdots ,N_{x}M_{x}$$

and try to find an approximate solution of

(54) $$s_{\phi }\left(v_{k}\right)+s_{\varphi }\left(v_{k}\right)=a_{x,k},k=1,2,\cdots ,N_{x}M_{x},$$

where $a_{x,k}=a_{x}\left(v_{k}\right)$. Thus, we utilize the KL divergence again in the following. Let $N_{x}$ be the sampling ratio. Let $V_{x}^{F}$ be an $N_{x}M_{x}\times N_{x}M_{x}$ DFT matrix and $V_{x}$ be an $N_{x}M_{x}\times M_{x}$ matrix defined by

(55) $$\begin{array}{cc} V_{x} & =V_{x}^{F}\left(\begin{array}{c} I_{M_{x}}\\ 0_{\left(N_{x}-1\right)M_{x}} \end{array}\right). \end{array}$$

We define ${\hat{a}}_{x,k}$ as

(56) $$\begin{array}{cc} {\hat{a}}_{x,k} & =s_{\phi }\left(v_{k}\right)+s_{\varphi }\left(v_{k}\right)\\ \; & =e_{k}^{T}V_{x}\phi \phi^{H}V_{x}^{H}e_{k}+e_{k}^{T}V_{x}\varphi \varphi^{H}V_{x}^{H}e_{k}. \end{array}$$

The optimization problem can be formulated as

(57) $$\begin{array}{cc} \min_{\left\{\rho_{i},\lambda_{i}\right\}} & \sum_{k=1}^{N_{x}M_{x}}a_{x,k}\log\frac{a_{x,k}}{{\hat{a}}_{x,k}}-\sum_{k=1}^{N_{x}M_{x}}a_{x,k}+\sum_{k=1}^{N_{x}M_{x}}{\hat{a}}_{x,k}. \end{array}$$

It can be proved that $\sum_{k=1}^{N_{x}M_{x}}{\hat{a}}_{x,k}=M_{x}$. Thus, the problem can be rewritten as

(58) $$\begin{array}{cc} \min_{\left\{\rho_{i},\lambda_{i}\right\}} & \sum_{k=1}^{N_{x}M_{x}}-a_{x,k}\log{\hat{a}}_{x,k}+d_{k}, \end{array}$$

where

(59) $$d_{k}=\sum_{k=1}^{N_{x}M_{x}}a_{x,k}\log a_{x,k}-\sum_{k=1}^{N_{x}M_{x}}a_{x,k}+M_{x}$$

is a constant not related to ϕ and φ or $\rho_{i}$ and $\lambda_{i}$. The above optimization problem is an unconstrained optimization problem, and thus a solution can be obtained by using the gradient method. Let g(ρ,λ) denote the objective function in Equation (58) as

(60) $$g\left(\rho ,\lambda \right)=\sum_{k=1}^{N_{x}M_{x}}-a_{x,k}\log{\hat{a}}_{x,k}+d_{k}.$$

In the following theorem, we present the gradients of *g* with respect to $\rho_{i}$ and $\lambda_{i}$, respectively.

**Theorem** **4.** *The gradients of the objective function g with respect to $\rho_{i}$ and $\lambda_{i}$ are given by*

(61) $$\begin{array}{cc} \;\;\frac{\partial g}{\partial \rho_{i}} & =\sum_{k=1}^{N_{x}M_{x}}-a_{x,k}{\hat{a}}_{x,k}^{-1}ℜ\left(j2\pi \phi^{H}e_{i}e_{i}^{T}V_{x}^{H}e_{k}e_{k}^{T}V_{x}\phi \right) \end{array}$$

(62) $$\begin{array}{cc} \;\;\frac{\partial g}{\partial \lambda_{i}} & =\sum_{k=1}^{N_{x}M_{x}}-a_{x,k}{\hat{a}}_{x,k}^{-1}ℜ\left(j2\pi \varphi^{H}e_{i}e_{i}^{T}V_{x}^{H}e_{k}e_{k}^{T}V_{x}\varphi \right). \end{array}$$

**Proof.**  The proof is provided in Appendix D. □

With the gradient provided in Theorem 4, the gradient method can be performed and summarized as

(63) $$\rho_{i}^{d+1}=\rho_{i}^{d}+\mu \frac{\partial g}{\partial \rho_{i}}$$

(64) $$\lambda_{i}^{d+1}=\lambda_{i}^{d}+\mu \frac{\partial g}{\partial \lambda_{i}},$$

where μ is the step size determined by the line search method to guarantee the convergence.

The steps of the proposed broad coverage precoder design are now summarized as follows:

1. Solve the problem (45) by using the projected gradient method provided in Equation (50) to obtain D. Obtain the vectors $d_{1},d_{2},\cdots ,d_{r}$ as the column of D.
2. Compute $f_{1},f_{2},\cdots ,f_{r}$ as $f_{1}=d_{2}^{†}$, $f_{2}=-d_{1}^{†}$, $f_{3}=d_{4}^{†}$, $f_{4}=-d_{3}^{†}$, $\cdots ,f_{r}=-d_{r-1}^{†}$.
3. Solve Equation (58) by using the gradient method provided in Equations (63) and (64) to obtain $\rho_{i}$ and $\lambda_{i}$. Generate ϕ and φ according to Equations (52) and (53).
4. Generate the precoding matrices as $P_{i}=d_{i}⊗\phi^{T}+f_{i}⊗\varphi^{T},\;i=1,2,\cdots ,r$.

## 4. Simulation Results

In this section, we provide simulation results to verify the analytic results of the proposed broad coverage precoder design. For simplicity, the target angle power spectra $a_{z}\left(u\right)$ and $a_{x}\left(v\right)$ are defined according to raised-cosine filters as

(65) $$a_{z}\left(u\right)=\left\{\begin{array}{c} 1,\;|u|\leq \alpha_{z}-\beta \\ 0,\;|u|\geq \alpha_{z}+\beta \\ \frac{1}{2}(1+\cos(\frac{\pi }{\beta }\left(|u\right|-\left(\alpha_{z}-\beta \right)))),\mathrm{otherwise} \end{array}\right.$$

and

(66) $$a_{x}\left(v\right)=\left\{\begin{array}{c} 1,\;|v|\leq \alpha_{x}-\beta \\ 0,\;|v|\geq \alpha_{x}+\beta \\ \frac{1}{2}(1+\cos(\frac{\pi }{\beta }\left(|v\right|-\left(\alpha_{x}-\beta \right)))),\mathrm{otherwise}, \end{array}\right.$$

where $\alpha_{x}$ and $\alpha_{z}$ are values related to horizontal and vertical coverage, and β is related to the roll-off factor. We set the number of the data streams *r* to 2 in all simulations.

**Figure 1:**
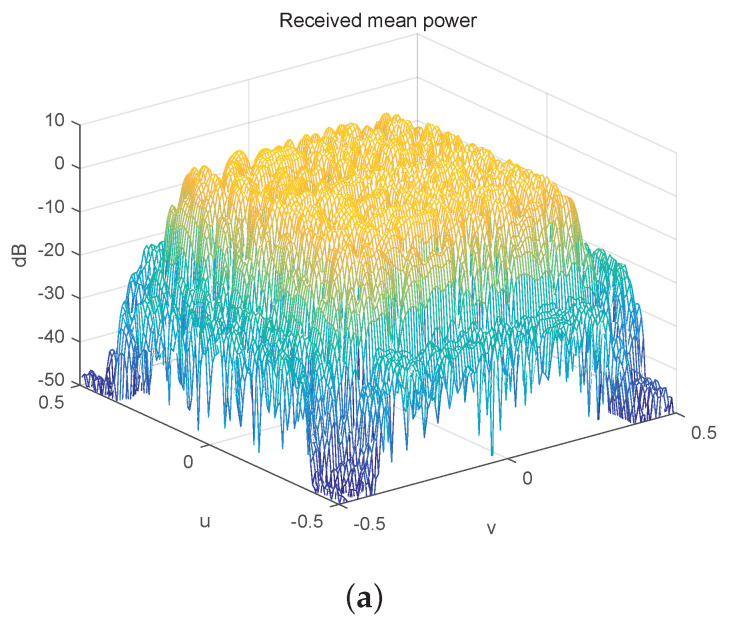

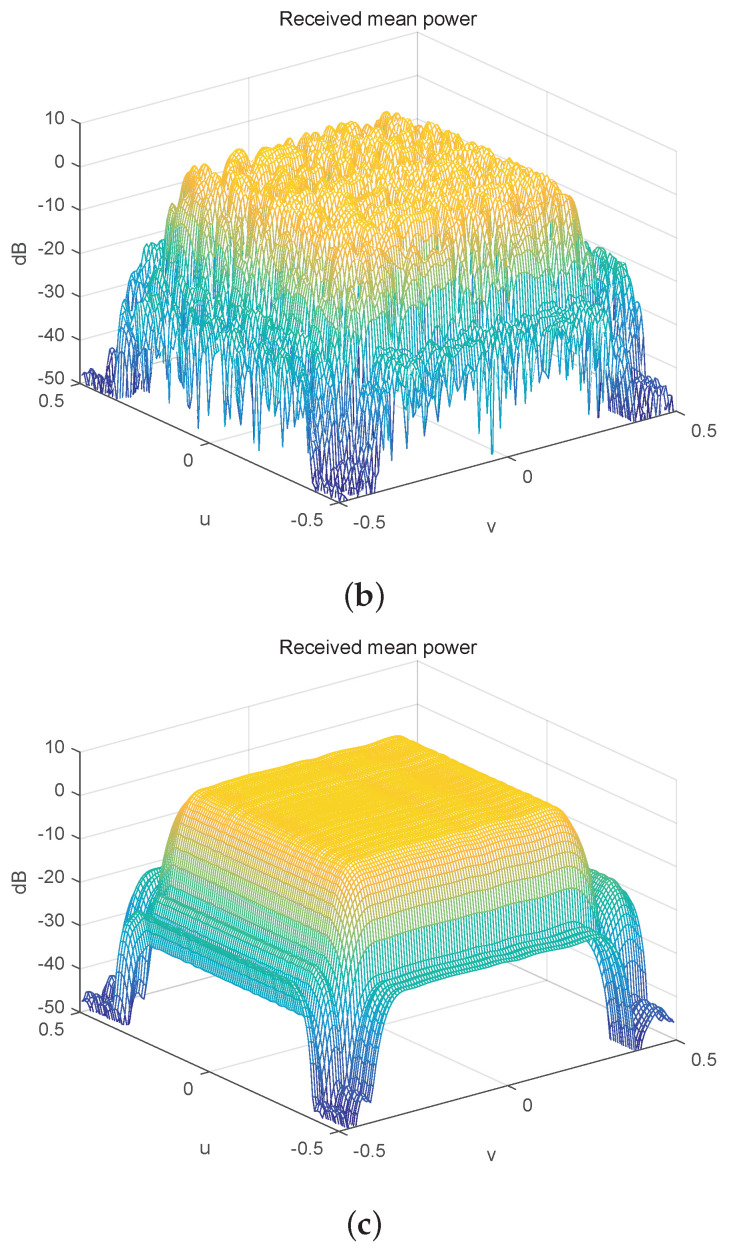
The anglepower spectrum of the proposed broad coverage precoder design. (**a**) The angle power spectrum of the 32 × 32 precoder $P_{1}$. (**b**) The angle power spectrum of the 32 × 32 precoder $P_{2}$. (**c**) The sum of the angle power spectrum of the 32 × 32 precoders $P_{1}$ and $P_{2}$.

In the first simulation, we set $M_{x}=M_{z}=32,\alpha_{x}=1/3$ and $\alpha_{z}=1/3$. We first obtain two sets of orthonormal sequences $\left\{d_{i}\right\},\left\{f_{i}\right\}$ with zero cross-correlation and an angle power spectrum close to $a_{z}\left(u\right)$ by using the method provided in Section 3.3. We then use the gradient method provided in Section 3.4 to generate the pair ϕ and φ with the sum of their angle power spectra being close to $a_{x}\left(v\right)$. Furthermore, we compute the broad coverage precoding matrices as $P_{i}=d_{i}⊗\phi^{T}+f_{i}⊗\varphi^{T},\;i=1,2$. The angle power spectra of the two precoding matrices are plotted in Figure 1a,b, and their sum is plotted in Figure 1c. From the figure, we observe that the shape of the generated power pattern of one stream has large perturbation, and whereas the sum of the generated power pattern of two streams has little perturbation. The sum is very close to that of the target power pattern $a_{z}\left(u\right)a_{x}\left(v\right)$. By using two streams, we are able to obtain a broad coverage precoding matrix whose angle power spectrum is very close to that of the target one.

**Figure 2:**
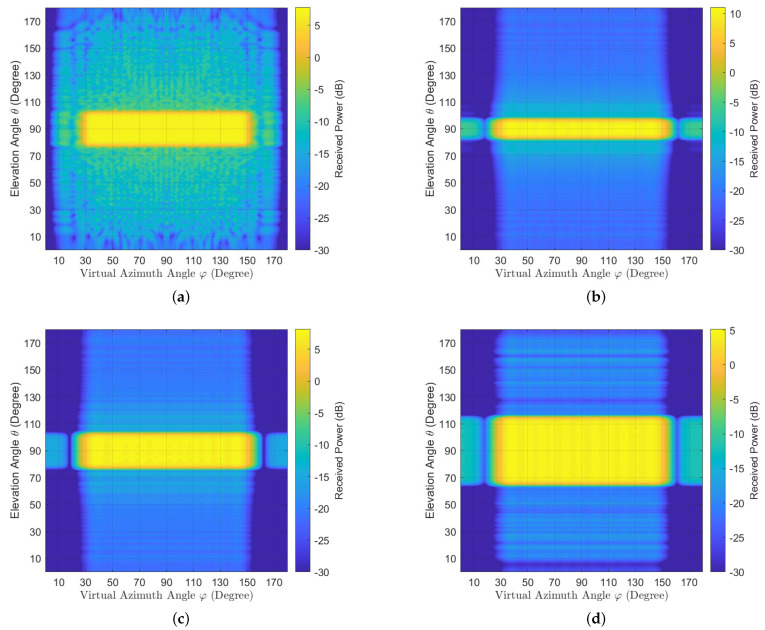
The radiationpower pattern generated by the precoders when r=2 and $M_{z}=64,M_{x}=32$. (**a**) The power pattern generated by the method from [18] with $\alpha_{z}=0.1$. (**b**) The power pattern generated by the proposed method with $\alpha_{z}=0.05$. (**c**) The power pattern generated by the proposed method with $\alpha_{z}=0.1$. (**d**) The power pattern generated by the proposed method with $\alpha_{z}=0.2$.

We then investigate the performance of the proposed broad coverage precoder in a 3D massive MIMO system. The simulation parameters are in Table 1. The base station of the considered system is equipped with a UPA with $M_{z}=64,M_{x}=32$ and is located at (0,0,25). The locations of the users are generated randomly in a 120 degree sector with radius R=200 m around the origin (0,0,0) at 1.5 m height. The downtilt degree is set as α=arctan(2h/R), where h=23.5 m. For convenience, we define the virtual horizontal angle as φ such that cosφ=cosϕsinθ. To cover the 120 degree sector, the value of $\alpha_{x}$ is set as $\alpha_{x}=\sqrt{3}/4$, whereas the value of $\alpha_{z}$ needs to be chosen according to the synchronization performance. In the simulations, we use $a_{z}=0.05,0.1$ and 0.2, the radiation power patterns of the proposed precoders are shown in Figure 2b–d. The range of the elevation angles becomes larger as the value of $a_{z}$ increases. Furthermore, all results show that the proposed method can generate broad coverage precoder having the radiation power pattern close to the target radiation power pattern. For comparison, the method from [18] is also used to generate the broad coverage precoder according to $a_{z}\left(u\right)a_{x}\left(v\right)$ with $\alpha_{x}=0.1$. The radiation power pattern of this case is plotted in Figure 2a. From the comparison of Figure 2a,c, it can be observed that the radiation power patterns of the two methods are nearly the same, but the proposed precoder design has lower leakage outside the desired coverage area than the method from [18]. This means that the proposed method causes less interference than the latter. The reason is that the dimensions of the optimization problems in the proposed precoder design are smaller, and thus it is easier to obtain good results.

**Figure 3:**
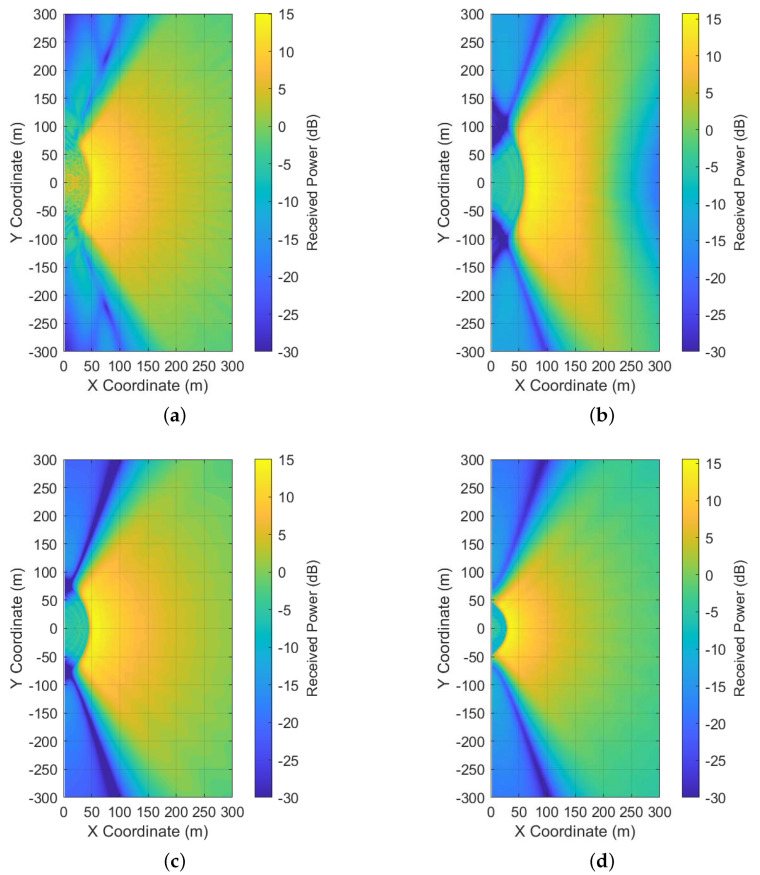
The receivedmean power in a 120-degree sector generated by the precoders when r=2 and $M_{z}=64,M_{x}=32$. (**a**) The power pattern generated by the method from [18] with $\alpha_{z}=0.1$. (**b**) The power pattern generated by the proposed method with $\alpha_{z}=0.05$. (**c**) The power pattern generated by the proposed method with $\alpha_{z}=0.1$. (**d**) The power pattern generated by the proposed method with $\alpha_{z}=0.2$.

Furthermore, to show the coverage of the proposed precoders in the 120-degree sector, the received mean powers of the proposed precoders at height 1.5 m are shown in Figure 3b–d. They show that the proposed precoder with $a_{z}=0.05$ has more evenly received mean power than the proposed precoders in the other two cases. However, a certain area that is far from the BS is not covered when $a_{z}=0.05$. Meanwhile, the received mean power of the proposed precoder with $a_{z}=0.2$ focuses more on the areas near the BS than those areas at the cell edge. Thus, the proposed precoder with $a_{z}=0.1$ provides better coverage than the proposed precoders from the other two cases. From the results of the received mean power, it seems the the proposed precoder with $a_{z}=0.1$ is preferred than the other two precoders. The received mean power of the method from [18] with $\alpha_{x}=0.1$ is plotted in Figure 3a. From the comparison of Figure 3a,c, we also observe that the proposed method has lower leakage outside the desired coverage area than the method from [18], and the two methods have nearly the same coverage.

**Figure 4:**
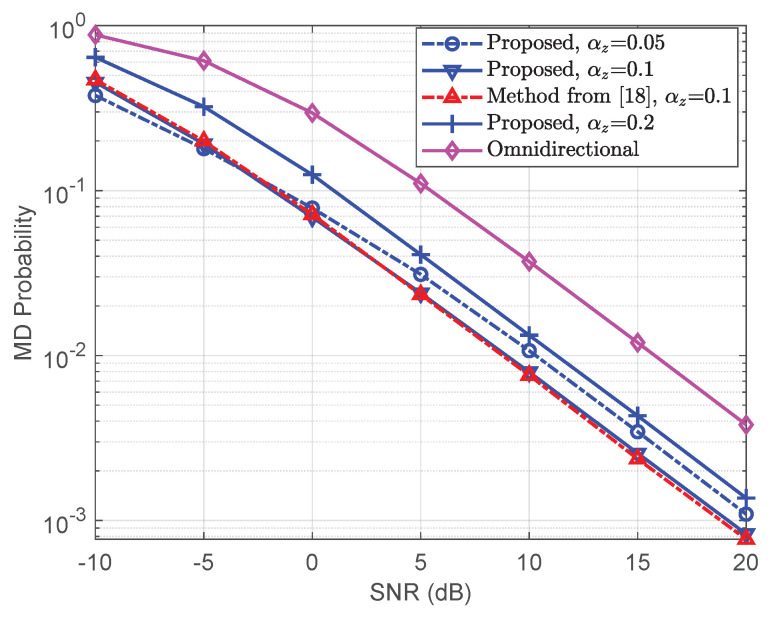
TheMD probabilities of the proposed precoder for the line of sight scenario.

Finally, we investigate the synchronization performance of the proposed precoders in the 120-degree sector. For simiplicity, we only consider the line of sight scenario. The users are randomly generated inside the sector, and then their channel coefficients are generated according to their location. The missed detection (MD) probability is one of the most important metrics that can characterize the synchronization performance. Thus, we use the MD probability to verify the synchronization performance of the proposed precoders. The MD probabilities of the proposed precoders are shown in Figure 4. The results of the omnidirectional precoder from [13] and the method from [18] are also provided as benchmarks. It shows that the proposed broad coverage precoder outperforms the omnidirectional precoder. This is because the omnidirectional precoder wastes part of its power by sending signals outside the sector. Among the three broad coverage precoders with different $\alpha_{z}$s, the proposed precoder with $\alpha_{z}=0.1$ has the best MD probability performance. This indicates that we can choose $\alpha_{z}=0.1$ for the considered 3D massive MIMO system to ensure a better synchronization performance. Meanwhile, the differences between the MD probabilities of the method from [18] and the proposed precoder are negligible. Thus, the proposed precoder can achieve nearly the same performance as the broad coverage precoder from [18] with much lower computational complexity.

## 5. Conclusions

In this paper, we proposed a broad coverage precoder design for massive MIMO with huge UPAs. The desired 2D angle power spectrum is assumed to be separable, and the per-antenna constant power constraint and semi-unitary constraint were considered. We proposed a method to construct the high-dimensional broad coverage precoding matrices from a set of orthonormal vectors and two vectors with certain properties. We also presented optimization methods to generate the set of orthonormal vectors and the pair of vectors, respectively. Finally, simulation results showed that the proposed broad coverage precoding matrices achieve nearly the same performance as the normal broad coverage precoder with much lower computational complexity.

The proposed broad coverage precoder is designed by assuming the required 2D angle power spectrum is separable. In practical use, the target 2D angle power spectrum might be inseparable. In this case, we might need to consider the problem of approximating the inseparable power spectrum with the separable power spectrum if we still want to use the proposed precoder design. We can also try to find a lower-complexity precoder design for the inseparable 2D angle power spectrum. However, it might be very hard to obtain a solution. Meanwhile, the proposed broad coverage precoder is for massive MIMO with UPAs. The design for other more advanced antenna configurations is also an interesting problem.

## Figures and Tables

**Table 1:** Simulation parameters for 3D massive MIMO.

Parameter	Value
Antenna configuration	$M_{z}=64,M_{x}=32$
BS location	(0, 0, 25)
Radius	200 m
Downtilt degree	arctan(2*h*/*R*)
Height of users	1.5 m

## Data Availability

Data sharing is not applicable to this article.

## Appendix A. A Lemma

**Lemma** **A1** (Theorem 1 in [13]). *Let $D=\{d_{1},d_{2},\cdots ,d_{r}\}$ and $F=\{f_{1},f_{2},\cdots ,f_{r}\}$ be two sets consisting of $M_{z}\times 1$ vectors. Let $D=\left[d_{1}\;d_{2}\;\cdots \;d_{r}\right]\in C^{M_{z}\times r}$ and $F=\left[f_{1}\;f_{2}\;\cdots \;f_{r}\right]\in C^{M_{z}\times r}$. We define the vector-valued functions $\tilde{d}\left(u\right)$ and $\tilde{f}\left(u\right)$ as*

(A1) $$\begin{array}{cc} \tilde{d}{\left(u\right)}^{T} & =v_{z}{\left(u\right)}^{T}D \end{array}$$

(A2) $$\begin{array}{cc} \tilde{f}{\left(u\right)}^{T} & =v_{z}{\left(u\right)}^{T}F. \end{array}$$

*If the aperiodic cross-correlation of D and F is zero, i.e.,*

(A3) $$\begin{array}{c} c_{D,F}\left(t\right)=0,t=-M_{z}+1,\cdots ,M_{z}-1 \end{array}$$

*then $\tilde{d}\left(u\right)$ and $\tilde{f}\left(u\right)$ are orthogonal for any u, i.e.,*

(A4) $$\begin{array}{c} \tilde{d}{\left(u\right)}^{H}\tilde{f}\left(u\right)=0,\;\forall u. \end{array}$$

## Appendix B. Proof of Theorem 1

The vector-valued function $q_{i}\left(u\right)$ is defined as

(A5) $$\begin{array}{cc} q_{i}{\left(u\right)}^{T} & =v_{z}{\left(u\right)}^{T}P_{i}\\ \; & =v_{z}{\left(u\right)}^{T}\left(d_{i}⊗\phi^{T}\right)+v_{z}{\left(u\right)}^{T}\left(f_{i}⊗\varphi^{T}\right). \end{array}$$

Let $Q\left(u\right)={\left[q_{1}\left(u\right)\;q_{2}\left(u\right)\;\cdots \;q_{r}\left(u\right)\right]}^{T}$, and we obtain

(A6) $$\begin{array}{cc} \; & \sum_{i=1}^{r}{\left|v_{z}{\left(u\right)}^{T}P_{i}v_{x}\left(v\right)\right|}^{2}=v_{x}{\left(v\right)}^{H}Q{\left(u\right)}^{H}Q\left(u\right)v_{x}\left(v\right). \end{array}$$

We define $\tilde{d}\left(u\right)$ and $\tilde{f}\left(u\right)$ as

$$\begin{array}{cc} \tilde{d}\left(u\right) & ={\left[{\tilde{d}}_{1}\left(u\right)\;{\tilde{d}}_{2}\left(u\right)\;\cdots \;{\tilde{d}}_{r}\left(u\right)\right]}^{T}\\ \tilde{f}\left(u\right) & ={\left[{\tilde{f}}_{1}\left(u\right)\;{\tilde{f}}_{2}\left(u\right)\;\cdots \;{\tilde{f}}_{r}\left(u\right)\right]}^{T}, \end{array}$$

where ${\tilde{d}}_{i}\left(u\right)=v_{z}{\left(u\right)}^{T}d_{i}$ and ${\tilde{f}}_{i}\left(u\right)=v_{z}{\left(u\right)}^{T}f_{i}$. We then have $Q\left(u\right)=\tilde{d}\left(u\right)⊗\phi^{T}+\tilde{f}\left(u\right)⊗\varphi^{T}$. Furthermore, we have

(A7) $$\begin{array}{cc} \;\;\;\;Q\left(u\right)v_{x}\left(v\right) & =\left(\tilde{d}\left(u\right)⊗\phi^{T}\right)v_{x}\left(v\right)+\left(\tilde{f}\left(u\right)⊗\varphi^{T}\right)v_{x}\left(v\right). \end{array}$$

According to the mixed product property of the Kronecker product, we then obtain

(A8) $$\begin{array}{cc} Q\left(u\right)v_{x}\left(v\right) & =\left(\tilde{d}\left(u\right)⊗\phi^{T}v_{x}\left(v\right)\right)+\left(\tilde{f}\left(u\right)⊗\varphi^{T}v_{x}\left(v\right)\right)\\ \; & =\tilde{d}\left(u\right)\left(\phi^{T}v_{x}\left(v\right)\right)+\tilde{f}\left(u\right)\left(\varphi^{T}v_{x}\left(v\right)\right). \end{array}$$

From Lemma A1, we see that $\tilde{d}\left(u\right)$ and $\tilde{f}\left(u\right)$ are orthogonal when the cross-correlation function $c_{D,F}\left(t\right)=0$. Using this condition, Equations (22), (A6) and (A8), we then have

(A9) $$\begin{array}{cc} \; & \sum_{i=1}^{r}{\left|v_{z}{\left(u\right)}^{T}P_{i}v_{x}\left(v\right)\right|}^{2}\\ \; & =e\left(u\right)|\phi^{T}v_{x}{\left(v\right)|}^{2}+e\left(u\right){\left|\varphi^{T}v_{x}\left(v\right)\right|}^{2}. \end{array}$$

From (23), we obtain the result Equation (25).

## Appendix C. Proof of Theorem 2

First, we obtain

(A10) $$\begin{array}{c} e_{k}^{T}P_{i}e_{l}=e_{k}^{T}\left(d_{i}⊗\phi^{T}\right)e_{l}+e_{k}^{T}\left(f_{i}⊗\varphi^{T}\right)e_{l}. \end{array}$$

According to the mixed product property of the Kronecker product, we then obtain

(A11) $$\begin{array}{cc} e_{k}^{T}P_{i}e_{l} & =\left(e_{k}^{T}d_{i}⊗\phi^{T}e_{l}\right)+\left(e_{k}^{T}f_{i}⊗\varphi^{T}e_{l}\right)\\ \; & =d_{ik}\phi_{l}+f_{ik}\varphi_{l}. \end{array}$$

Furthermore, we have

(A12) $$\begin{array}{cc} \sum_{i=1}^{r}{\left|e_{k}^{T}P_{i}e_{l}\right|}^{2} & =\sum_{i=1}^{r}\left(\right|d_{ik}|{}^{2}|\phi_{l}|{}^{2}+|f_{ik}|{}^{2}|\varphi_{l}|{}^{2}). \end{array}$$

Since $\sum_{i=1}^{r}|d_{ik}{|}^{2}=\sum_{i=1}^{r}{\left|f_{ik}\right|}^{2}=1$, and the fact that one of $|\phi_{l}{|}^{2}$ and $|\varphi_{l}{|}^{2}$ must be zero and the other equal one, we have

(A13) $$\begin{array}{cc} \sum_{i=1}^{r}{\left|e_{k}^{T}P_{i}e_{l}\right|}^{2} & =1. \end{array}$$

From

(A14) $$|p_{i,kl}{|}^{2}={\left|e_{k}^{T}P_{i}e_{l}\right|}^{2}$$

we then obtain the result in the theorem.

## Appendix D. Proof of Theorem 4

From the chain rule, we have

(A15) $$\frac{\partial g}{\partial \rho_{i}}=\sum_{k=1}^{N_{x}M_{x}}-a_{x,k}{\hat{a}}_{x,k}^{-1}\frac{\partial {\hat{a}}_{x,k}}{\partial \rho_{i}}.$$

From the definition of ${\hat{a}}_{x,k}$, we have

(A16) $$\begin{array}{cc} \frac{\partial {\hat{a}}_{x,k}}{\partial \rho_{i}}\; & =\frac{\partial e_{k}^{T}V_{x}\phi \phi^{H}V_{x}^{H}e_{k}}{\partial \rho_{i}}\\ \; & =\frac{e_{k}^{T}V_{x}e_{i}\left(\partial e_{i}^{T}\phi \right)\phi^{H}V_{x}^{H}e_{k}}{\partial \rho_{i}}\\ \; & \;\;+\frac{e_{k}^{T}V_{x}\phi \left(\partial \phi^{H}e_{i}\right)e_{i}^{T}V_{x}^{H}e_{k}}{\partial \rho_{i}}. \end{array}$$

According the definition of ϕ in Equation (52), we obtain

(A17) $$\frac{\partial e_{i}^{T}\phi }{\partial \rho_{i}}=-j2\pi e_{i}^{T}\phi$$

and

(A18) $$\frac{\partial \phi^{H}e_{i}}{\partial \rho_{i}}=j2\pi \phi^{H}e_{i}.$$

Substituting Equations (A17) and (A18) into Equation (A16), we obtain

(A19) $$\begin{array}{cc} \frac{\partial {\hat{a}}_{x,k}}{\partial \rho_{i}}\; & =-j2\pi e_{k}^{T}V_{x}e_{i}e_{i}^{T}\phi \phi^{H}V_{x}^{H}e_{k}\\ \; & \;\;\;\;+j2\pi e_{k}^{T}V_{x}\phi \phi^{H}e_{i}e_{i}^{T}V_{x}^{H}e_{k}\\ \; & =ℜ(j2\pi \phi^{H}e_{i}e_{i}^{T}V_{x}^{H}e_{k}e_{k}^{T}V_{x}\phi ). \end{array}$$

Then, we obtain

(A20) $$\;\;\frac{\partial g}{\partial \rho_{i}}=\sum_{k=1}^{N_{x}M_{x}}-a_{x,k}{\hat{a}}_{x,k}^{-1}ℜ\left(j2\pi \phi^{H}e_{i}e_{i}^{T}V_{x}^{H}e_{k}e_{k}^{T}V_{x}\phi \right).$$

Finally, we can obtain the gradient of *g* with respect to $\lambda_{i}$ in a similar way.
